# Geometry and Evolution of the Cangdong Sag in the Bohai Bay Basin, China: Implications for Subduction of the Pacific Plate

**DOI:** 10.1038/s41598-017-15759-x

**Published:** 2017-11-13

**Authors:** Liang Luo, Jiafu Qi, Hongxiang Li, Yueqi Dong, Shuai Zhang, Xichen Zhang, Xiaoxia Yu, Lingyan Luo

**Affiliations:** 1 State Key Laboratory of Petroleum Resources and Prospecting, China University of Petroleum, Beijing, 102249 China; 2 College of Geosciences, China University of Petroleum, Beijing, 102249 China; 3 Exploration and Development Research Institute of Dagang Oilfield Company, Tianjin, 300280 China

## Abstract

The Cangdong Sag is a complex Cenozoic rift basin at the center of the Bohai Bay Basin. Cenozoic structures in the Cangdong Sag can be subdivided into the Cangdong Fault System in the west and the Xuxi Fault System in the east. The geometry of the boundary faults varies along the axes of half-grabens. According to the cross-sectional strata geometry, unconformity and planar structural pattern, the Cenozoic structural evolution of the Cangdong Sag can be divided into four distinct stages: (1) major Paleocene initial rift, (2) latest Paleocene–early Eocene intensive rift, (3) late Eocene–Oligocene strike–slip superimposed rift, and (4) Neogene to present-day post-rift depression. The extensional deformation was mainly derived from horizontal stress induced by the upwelling of asthenosphere. The strike–slip structure of the Cangdong Sag provides important information related to the subduction of the Western Pacific Plate. It was found that the strike–slip movement of the southern Xuxi Fault Zone was activated during the deposition of the third member of Shahejie Formation to the Dongying Formation; therefore, ~43 Ma probably marks the time when the Western Pacific Plate initially changed its subduction direction from northwest to nearly west.

## Introduction

**Figure 1:**
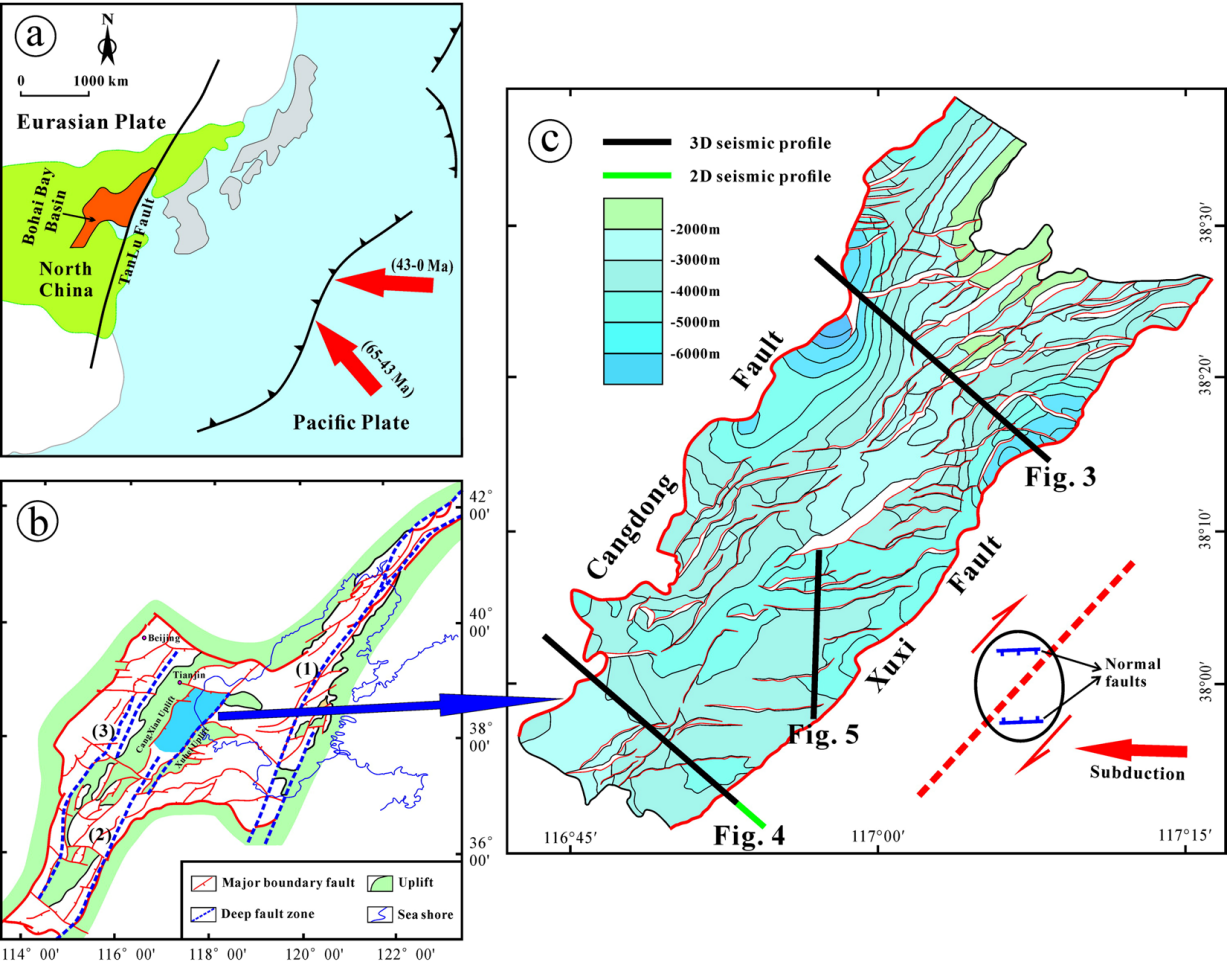
(**a**) Regional tectonic framework and location of the Bohai Bay Basin (modified from Qi & Yang^2^). (**b**) Cenozoic structural map of the Bohai Bay Basin (modified from Qi & Yang^2^) and the location of the Cangdong Sag in the Huanghua Depression. (1) Shenyang–Weifang Fault Zone; (2) Huanghua–Dezhou–Dongming Fault Zone; (3) Baxian–Shulu–Handan Fault Zone. (**c**) Structural map of the bottom of Ek^2^ of the Cangdong Sag. Figure 1c was generated using the LandMark software (Version 2003) (https://www.landmark.solutions/).

According to the distribution of Paleogene deposits, the Bohai Bay Basin in the East China can be subdivided into eight depressions and five uplifts^1,2^. Generally, the depressions consist of several sags that are filled with relatively thick Paleogene deposits, separated by uplifts that are covered with thinner Paleogene deposits. The depressions and sags are commonly bounded by normal faults on one or several sides. The Cangdong Sag belongs to the Huanghua Depression and is located in Tianjin and Hubei. The sag is bordered by the Cangxian Uplift to the west and the Xuhei Uplift to the east (Fig. 1b).

**Figure 2:**
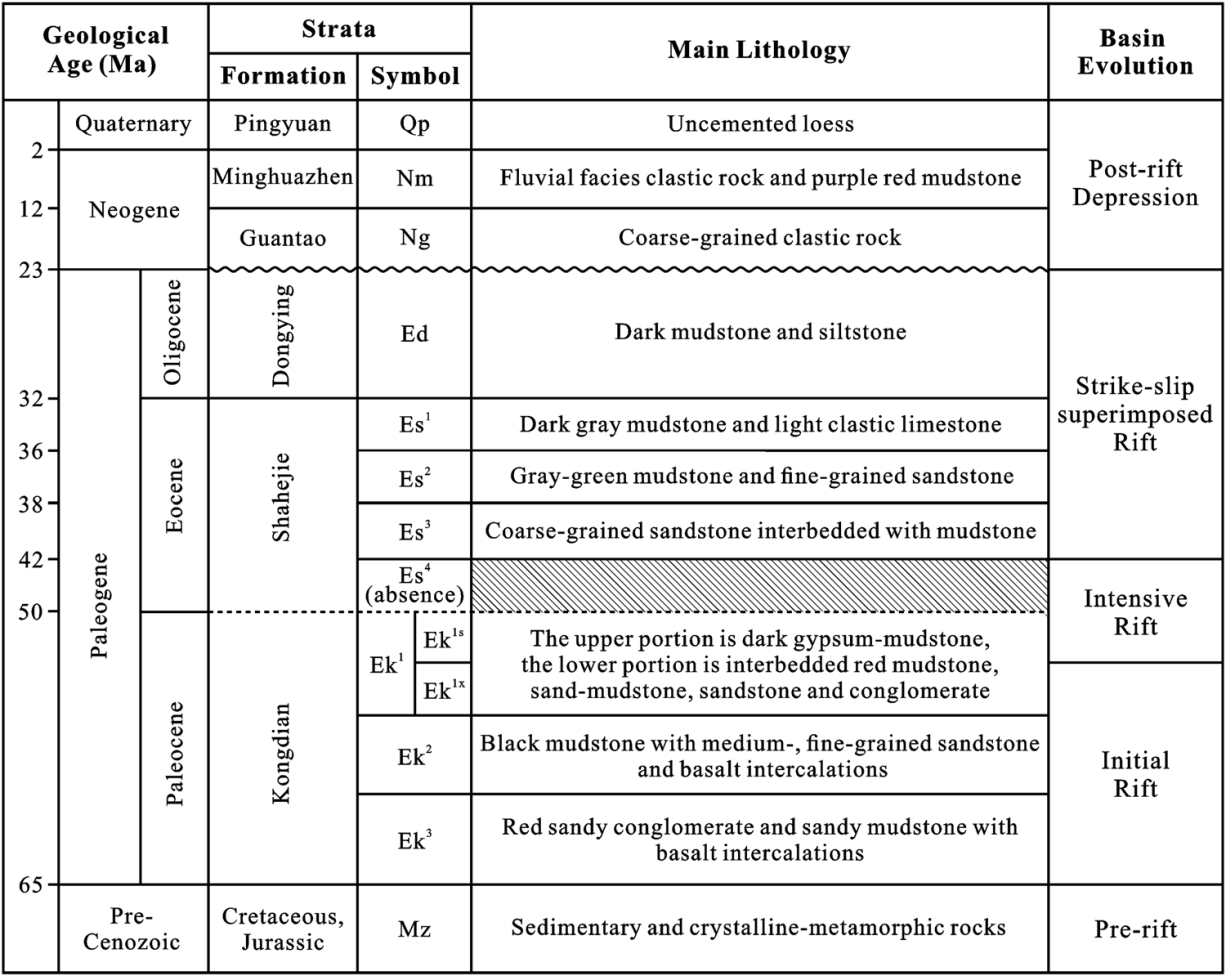
Cenozoic strata and basin evolution of the Cangdong Sag. The map was produced using the CorelDRAW (Version X7) (http://www.corel.com/cn/).

The Cangdong Sag is a superimposed basin formed during the Mesozoic and Cenozoic. The prototype of Mesozoic basin is not clearly identified. The Cangdong Sag developed as a part of the Bohai Bay Basin since the Paleogene. The strata sequences are characteristic of a typical depression basin, that is, comprising early-stage faults with sediments of different thickness and late-stage thermal subsidence with widely distributed sediments. Petroleum exploration data have revealed that the sag can be subdivided into three tectonostratigraphic levels^2^ (Fig. 2). The pre-Cenozoic basement, which is dissected by many faults, comprises sedimentary and crystalline-metamorphic rocks. The composition of the Paleogene sequences is lacustrine, with alluvial sedimentary rocks intercalated with oil shale. The Paleogene strata include the Kongdian Formation (subdivided into three members, Ek^1^, Ek^2^, and Ek^3^, of which Ek^1^ can be further subdivided into the lower and upper portions, Ek^1x^ and Ek^1s^), the Shahejie Formation (subdivided into four members, Es^1^, Es^2^, Es^3^, and Es^4^), and the Dongying Formation, which is deposited on the faulted basement. Because of large-area regression in the Huanghua depression after transgression during the deposition of Kongdian Formation, Es^4^ is absent in the Cangdong Sag, and the contact relationship between the Es^3^ and the Kongdian Formation is a disconformity^2^. The Neogene–Quaternary sequences (Guantao Formation, Minghuazhen Formation and Pingyuan Formation), which are dominated by fluvial and alluvial fan sediments, are deposited over the whole basin and cover the syn-rift successions, which are separated by a regional unconformity above the Dongying Formation^3,4^.

The formation and development mechanisms of the Bohai Bay Basin and its subbasins during the Cenozoic are still highly debated^2,5–17^. Nevertheless, according to the special tectonic location of the Bohai Bay Basin on the Eurasian Plate (Fig. 1a), it is widely accepted that the Cenozoic structure is related to the surrounding plate movements, including that of the Pacific Plate and Indian Plate^2,9,10,12,15,18–22^. Therefore, the structural architecture and kinematics of the Cangdong Sag should provide important information related to the adjacent plate motion. In this paper, we use high-quality 3D and 2D seismic data to discuss the key geological issues related to the geometry, evolution, and geodynamics of the Cangdong Sag. We also analyze the strike–slip process of the Cangdong Sag and discuss its relation to the subduction of the Western Pacific Plate beneath the Eurasian Plate.

## Method

**Figure 3:**
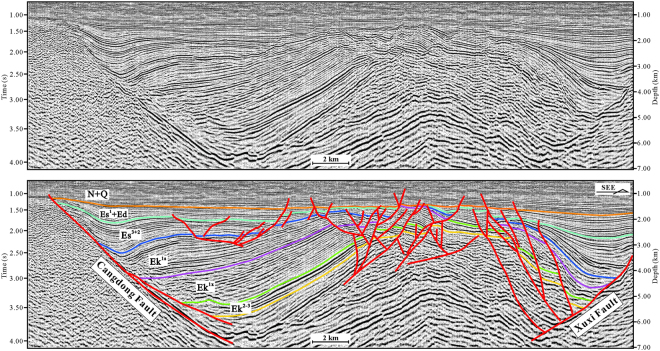
Seismic profile in the northern region of the Cangdong Sag (for line location, see Fig. 1c). The seismic profile was interpreted using the LandMark software (Version 2003) (https://www.landmark.solutions/).

**Figure 4:**
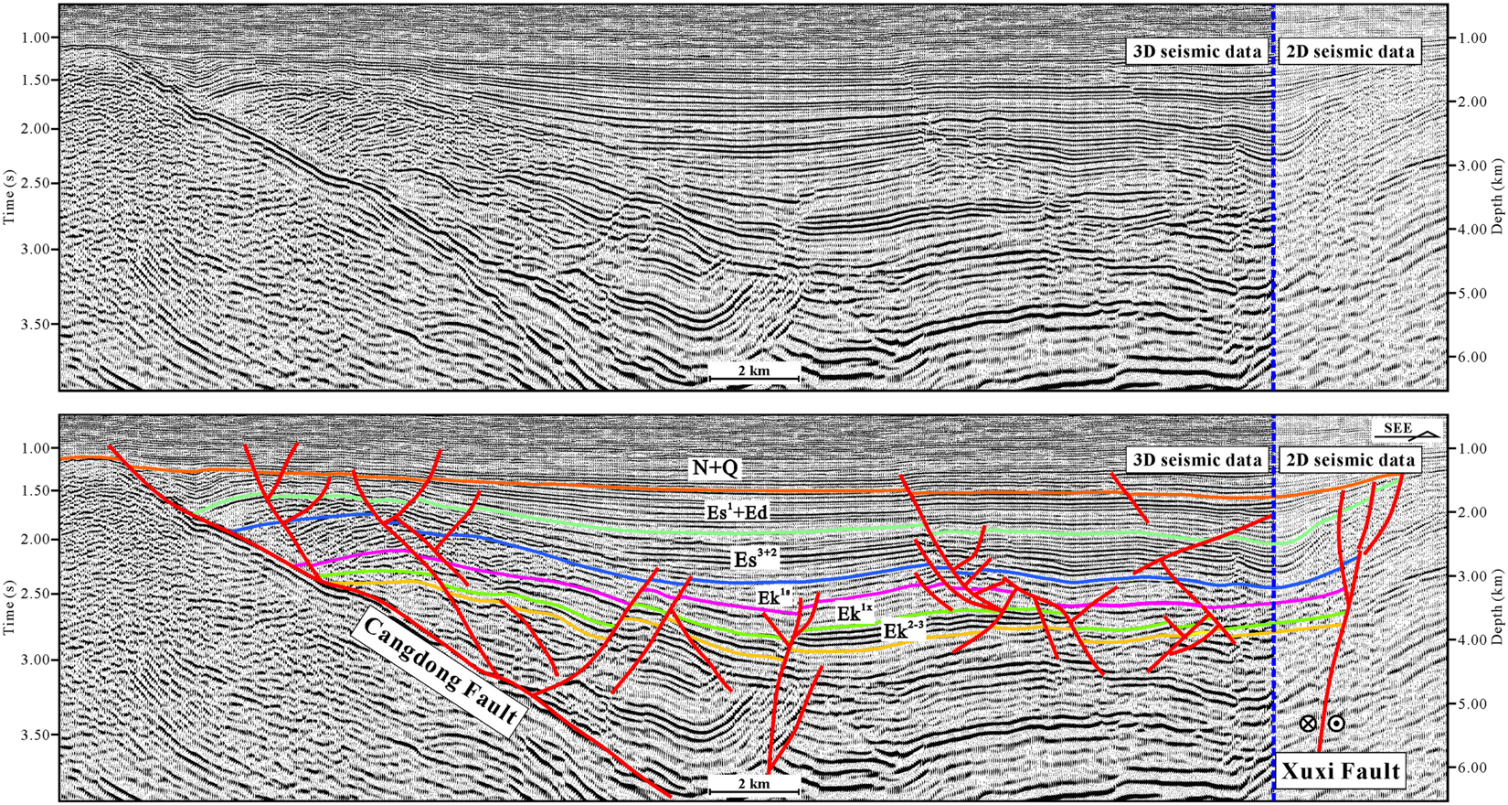
Seismic profile in the southern region of the Cangdong Sag (for line location, see Fig. 1c). The seismic profile was interpreted using the LandMark software (Version 2003) (https://www.landmark.solutions/).

**Figure 5:**
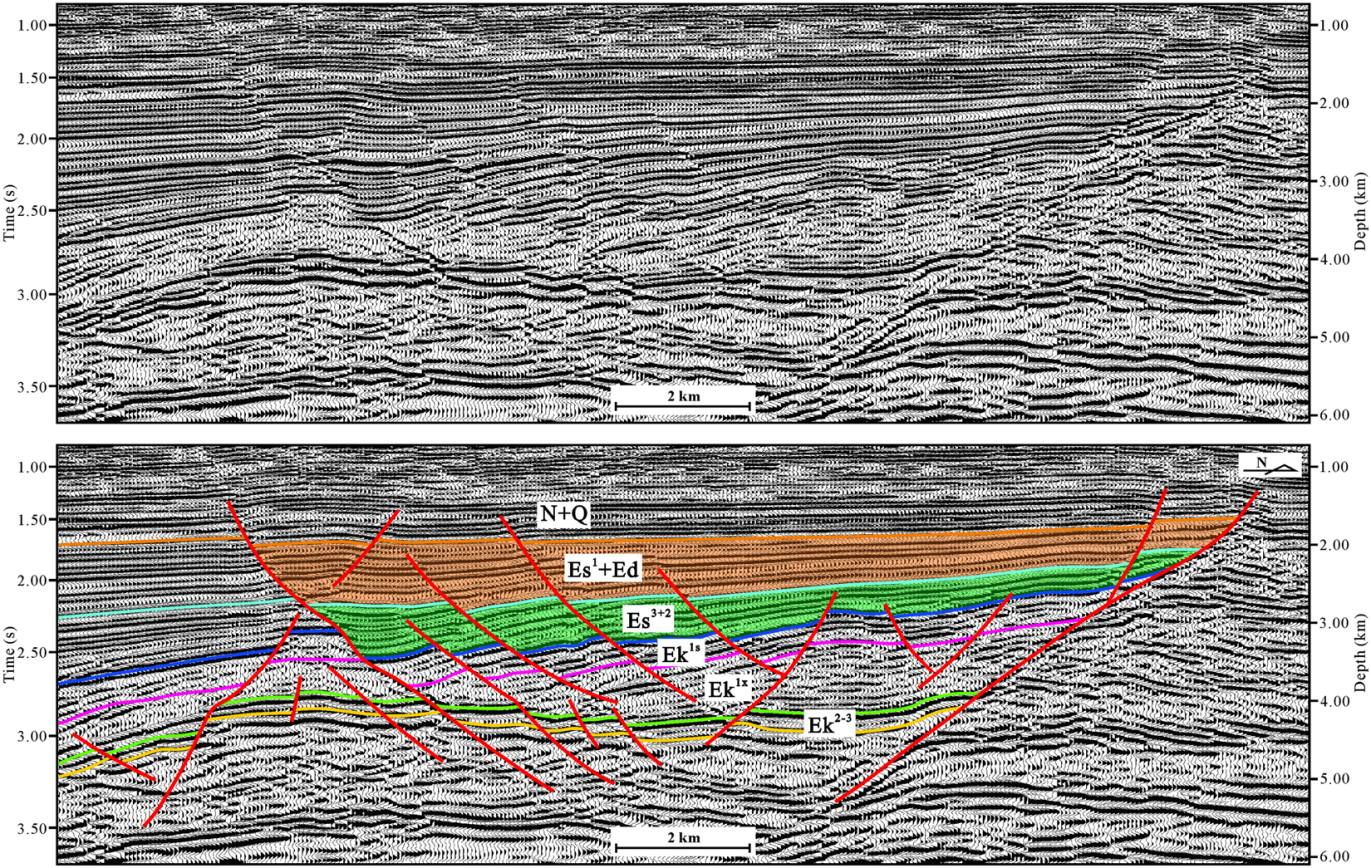
Seismic profile perpendicular to the strike of subordinate en echelon normal faults oblique to the Xuxi Fault in the Cangdong Sag (for line location, see Fig. 1c). The seismic profile was interpreted using the LandMark software (Version 2003) (https://www.landmark.solutions/).

Our structural map and cross-sections are based on an integrated analysis of a substantial database, consisting of 3D and 2D seismic reflection data. The seismic data were obtained from the Exploration and Development Research Institute of Dagang Oilfield Company. Through redefinition of the coordinate of shot point and receiver point, denoise, amplitude compensation, deconvolution, velocity analysis and statics, common-midpoint gather (CMP gather) was achieved from the demultiplex field data. Velocity in depth domain was converted from RMS velocity field. Common reflector gather (time or depth) can be migrated from CMP gather and velocity field (time or depth). Finally, prestack time migration section and prestack depth migration section were stacked from common reflector gather (time and depth respectively). Similar processing flows have been effective for crustal-scale reflection seismic studies^23–25^. According to synthetic seismograms, six layers (the bottoms of the Ek^3^, Ek^1x^, Ek^1s^, Es^3^, Es^1^ and Ng) were calibrated (Figs 3, 4 and 5). The 3D seismic data were interpreted at a 400 m × 400 m density.

### Geometry and Evolution of the Cangdong Sag

The typical structure of a rift basin is an extensional fault system. Major normal faults, synthetic or antithetic secondary faults, and transverse transfer faults may be strongly or weakly linked with, or terminated by, detachment faults^26^. The linked extensional fault systems play an important role in controlling the deposition of syn-rifting sequences^27^. The major normal faults within allochthons are regarded as “active” structures during basin evolution, whereas transverse and oblique structures are regarded as “passive” structures^28,29^, and all of these structures should be considered structural elements of extensional fault systems^26,30^. Generally, the major boundary faults of half-grabens or asymmetric full grabens are basement-involved normal faults, and the synthetic and/or antithetic secondary faults include both basement-involved and cover-transected normal faults at different scales.

The structure and deposition of the Cangdong Sag were mainly controlled by two basin-margin NE–trending extensional faults: Cangdong Fault to the west and Xuxi Fault to the east (Fig. 1c). On the basis of the distribution, attitude, and relationship between the faults and the sag, the Cenozoic structures within the Cangdong Sag can be subdivided into the Cangdong Fault System and Xuxi Fault System. Along the axes of half-grabens, the geometry of boundary faults is variable (Figs 3 and 4). In the northern part of the Cangdong Sag, the Cangdong and Xuxi Faults are characterized by a listric shape and a detachment within the basement. Although in cross-section the two faults each display a planar normal structure in the southern region, the Cangdong Fault is gently dipping and the Xuxi Fault is steep. The majority of faults in the Cangdong Fault System are NE–ENE–trending (Fig. 1c), with a series of rotational planar secondary faults forming a domino-style half-graben system above the deep Cangdong detachment fault (Figs 3 and 4). The secondary faults are mainly parallel to the Xuxi Fault in the northern Xuxi Fault System (Fig. 1c). The consistent regional structural pattern allows us to infer a right-lateral strike–slip for the southern Xuxi Fault System. The structural association of the steep basement-involved Xuxi Fault and the cover-transected relatively low-angle normal faults displays a negative “flower structure”^31^ in the seismic cross-section (Fig. 4). In addition, the structural map of the bottom of Ek^2^ indicates that a set of subordinate en echelon normal faults occur in a left-stepping pattern and oblique to the Xuxi Fault (Fig. 1c). The azimuths of these structures are consistent with a right-lateral strike–slip strain ellipse^32^ (Fig. 1c).

Generally, strata geometry is controlled by the extensional fault activity in the rift basin. The growth strata become thinner away from the extensional fault, whereas the thickness of pre-growth strata is consistent. The pre-Cenozoic can be regarded as the pre-rift Megasequence^13^. The regional unconformity between the Paleogene and Neogene deposits represents an important tectonic event that marks the termination of the rift stage and the beginning of post-rift depression stage^2^. Some of the faults cut into the Neogene sequences; however, they were not growth faults and had no influence on deposition during the Neogene (Figs 3 and 4). During the deposition of Ek^3^ to Ek^1x^, strata thickness was relatively consistent (Figs 3 and 4), indicating that the deposition sequences were not dominantly controlled by faults. This stage can be regarded as an initial rift. The syn-depositional extensional faults were mainly developed from the Ek^1s^ to the Dongying Formation, and the sequences within the sag commonly display a wedge-shaped geometry (Figs 3 and 4). This can be interpreted as an intensive rift basin developed in the hanging walls of detachment faults. The wedge-shaped geometry is more visible in the northern Cangdong Sag (Figs 3 and 4), which indicates that rifting was concentrated in the northern region.

One representative seismic profile approximately perpendicular to the strike of subordinate en echelon normal faults oblique to the Xuxi Fault was chosen and interpreted (Figs 1c and 5). The wedge-shaped geometry of the Es^3^ to the Dongying Formation indicates that the typical characteristics of these faults and the right-lateral movement of the Xuxi Fault developed during the late Eocene–Oligocene. Although the strike–slip system affected local deposition in the southern Cangdong Sag, it did not produce a visible pull-apart basin. The right-lateral strike–slip characteristic was superimposed on the extensional Xuxi Fault Zone during the late Eocene–Oligocene, and this can be interpreted as a strike–slip superimposed rift basin.

**Figure 6:**
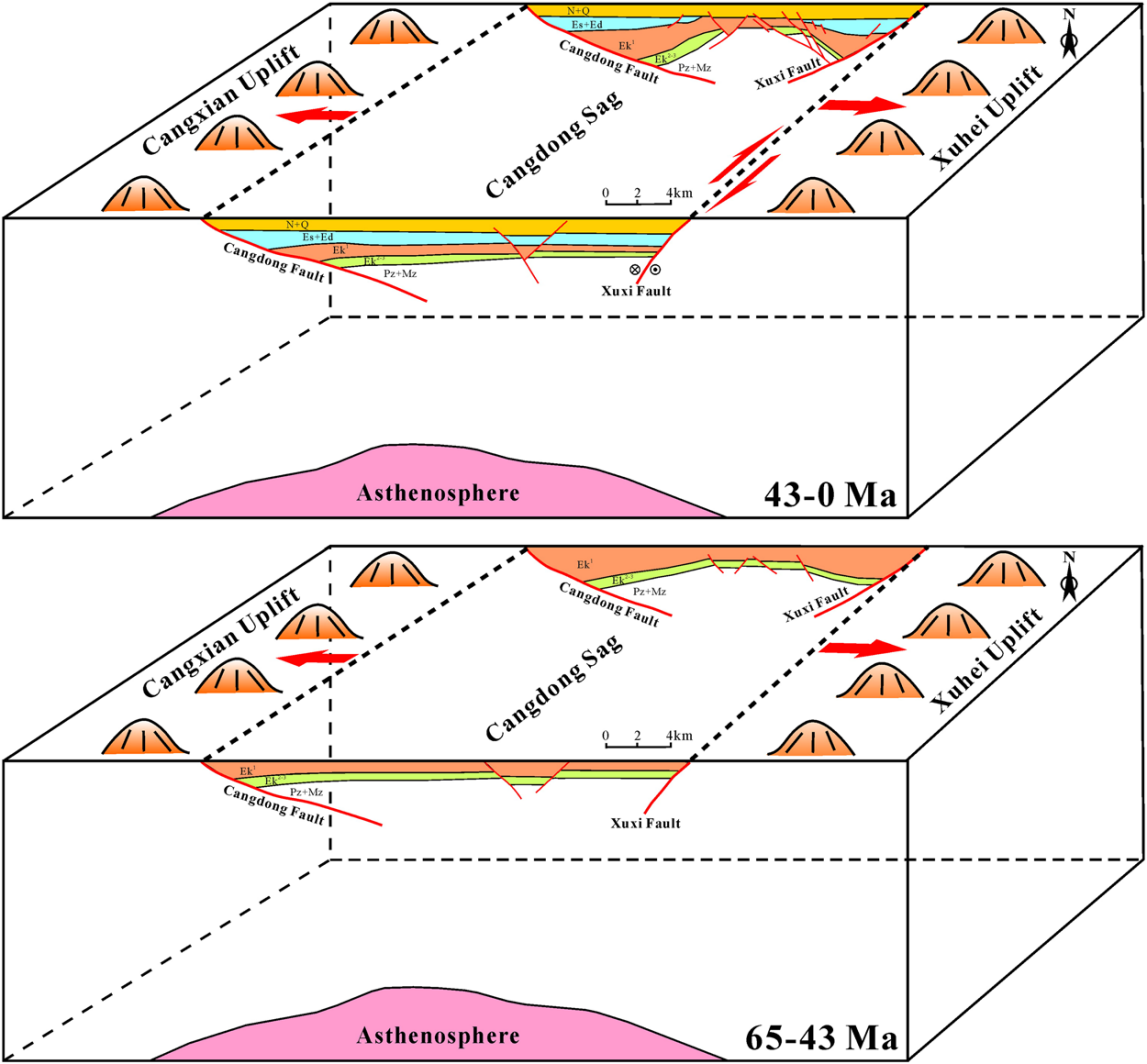
The sketch showing dynamic mechanism of the Cenozoic Cangdong Sag. The map was produced using the CorelDRAW (Version X7) (http://www.corel.com/cn/).

Based on the above description, the depression process of the Cangdong Sag continued from Paleocene to Quaternary. However, different from common rift basin, which is characterized by two stages of rift and post-rift thermal subsidence, the Cangdong Sag shows a strike–slip activity during the deposition of the Es^3^ to the Dongying Formation. Therefore, the Cenozoic structural evolution of the Cangdong Sag can be divided into four distinct stages (Figs 2 and 6): (1) major Paleocene initial rift, (2) latest Paleocene–early Eocene intensive rift, (3) late Eocene–Oligocene strike–slip superimposed rift, and (4) Neogene to present-day post-rift depression.

## Discussion and Conclusion

Extensional faults are widespread and penetrate the entire Cangdong Sag, whereas strike–slip faults with their associated structural elements are concentrated in the southern Xuxi Fault System. The major extensional faults were initiated in the latest Paleocene, and extensional deformation essentially ceased by the end of the Oligocene. The right-lateral strike–slip system was evidently initiated in the late Eocene, and strike–slip deformation maybe continued into the Neogene or even the Quaternary^13^. As the Cenozoic strike–slip fault coincides with the extensional fault in terms of strikes, the strike–slip and extensional deformations are not compatible in a theoretically unified regime^21^. Therefore, Cenozoic structural deformation in the Cangdong Sag is a result of superposition of multiple processes.

The dynamic mechanism of Cenozoic rifting in the Bohai Bay Basin is debatable. As presented by various researchers^2,9,10,14,15,17,18,20,22,33,34^, it might be derived from (i) subduction of the Pacific Plate beneath the Eurasian Continent, (ii) eastward lateral escape due to collision between the Indian Plate and the Eurasian Plate, or (iii) thermal diapirism beneath the continental plate. Driving of the extensional stress field in the plate interior by the inter-motion of plates along their boundary is difficult, especially in the region distant from the plate boundary, therefore, this probably originated from the bottom of lithosphere^35^. We are inclined to accept the dynamic model proposed by Qi & Yang^2^. The active rifting process in the Cangdong Sag was succeeded by a passive shearing process along the deep fracture zone during the Cenozoic. As the lithosphere warmed due to the asthenospheric upwelling, expansion of the lithosphere counteracted the effect of the regional compression force transmitted from the plate boundary, thereby inducing lithosphere extension (Fig. 6). One prominent evidence is that intensive basic volcanism was developed during the Ek^1^ to Ed in the Huanghua Depression, which can be the thermal origin.

The strike–slip fault system in the Cangdong Sag probably resulted from a regional stress field induced by adjacent plate movement, which can be related to the subduction of the Western Pacific Plate to the east or the collision of the Indian Plate to the west. There are three regional NE–NNE–trending right-lateral strike–slip fault zones in the Bohai Bay Basin: Shenyang–Weifang Fault Zone, Huanghua–Dezhou–Dongming Fault Zone, and Baxian–Shulu–Handan Fault Zone (from east to west) (Fig. 1b). The Shenyang–Weifang Fault Zone forms part of the Tanlu Deep Fault Zone, which is the most important fault zone in East China. The Tanlu Fault was initiated with left-lateral strike–slip at least during the Mesozoic^36,37^, and the Cenozoic activity transferred from transtension to transpression in the Liaohe Western Depression^21,38–40^. The deep fault zones gradually decrease in size, intensity, and continuity from east to west^2^, indicating that the force was generated from the east. In addition, the Bohai Bay Basin occupies an intracontinental position approximately 2,000 km from the Himalayas. Therefore, we suggest that the driving force for strike–slip in the southern Cangdong Sag originated from the subduction of the Western Pacific Plate beneath the Eurasian Plate.

Owing to the Hawaiian–Emperor bend, it is widely recognized that the Western Pacific Plate changed its subduction direction from northwest to nearly west in the Cenozoic (Fig. 1a); however, the timing of this change is controversial, with estimates of about 43 Ma^41–45^ and about 50 Ma in some recent studies^46,47^. The right-lateral movement of the NE–trending strike–slip fault zone in the Cangdong Sag was triggered by the shear stress component of the nearly E–W subduction of the Western Pacific Plate relative to the Eurasian Plate (Fig. 1c). Therefore, the strike–slip fault system may provide important information with which to confirm the time when the subduction direction of the Western Pacific Plate changed. Based on the strata geometry adjacent to the en echelon normal faults, strike–slip faulting was initiated during the deposition of Es^3^ (approximately 42–38 Ma) (Fig. 6), which indicate that the Western Pacific Plate changed its subduction direction probably at about 43 Ma (Fig. 1a).

## Electronic supplementary material

Supplementary Information
