# Supplementary material for: Geometry and Evolution of the Cangdong Sag in the Bohai Bay Basin, China: Implications for Subduction of the Pacific Plate
Source: Sci Rep. 2017 Nov 13;7:15393. doi: 10.1038/s41598-017-15759-x (PMC5684199; doi:10.1038/s41598-017-15759-x)

Supplementary Information

Geometry and Evolution of the Cangdong Sag in the Bohai Bay Basin, China: Implications for Subduction of the Pacific Plate

Liang Luo[1,2*], Jiafu Qi [1,2], Hongxiang Li [3], Yueqi Dong [3], Shuai Zhang [1,2], Xichen Zhang [1,2], Xiaoxia Yu [1,2] & Lingyan Luo [1,2]

[1]State Key Laboratory of Petroleum Resources and Prospecting, China University of Petroleum, Beijing 102249, China

[2]College of Geosciences, China University of Petroleum, Beijing 102249, China

[3]Exploration and Development Research Institute of Dagang Oilfield Company, Tianjin 300280, China

[*]Corresponding author. E-mail address: luoliang1225@163.com

This files include Supplementary Table S1 and Figure S1.

Table S1: Acquisition Parameters for the Reflection Seismic Data.

Parameter	Unit
Recording system	SERCEL 428XL
Spread geometry	28 lines, 10 shots, 360 channels
No. of live channels	10080
Maximum offset	4547 m
Nominal CMP fold	252
Receiver spacing	20 m
Source interval	20 m
Recording length	6 s
Sampling rate	1 ms
Geophone frequency	5 Hz
Geophone per set	Single
Sweeps	10
Shots	10

Figure S1: Example of how the seismic section (part of Figure 4 in the manuscript) was processed and how the time-depth conversion was achieved. (a) original single shot record; (b) prestack time migration seismic section; (c) prestack depth migration seismic section.

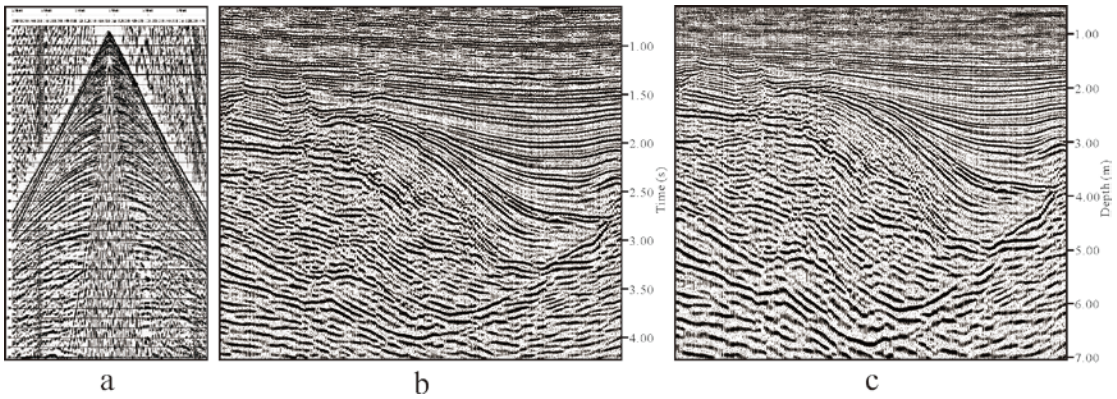

Demultiplex field data → Redefine the coordinate of shot point and receiver point → Denoise → Amplitude compensation → Deconvolution → velocity analysis and statics → CMP Gather (common-midpoint gather) **(Figure S1a)**

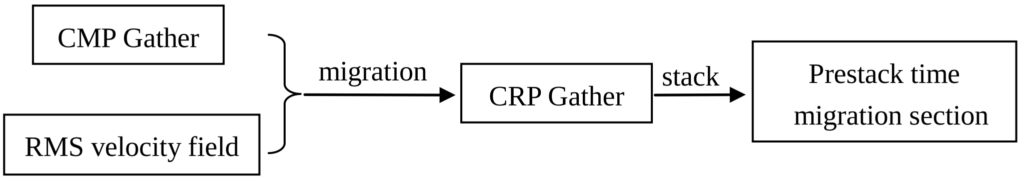

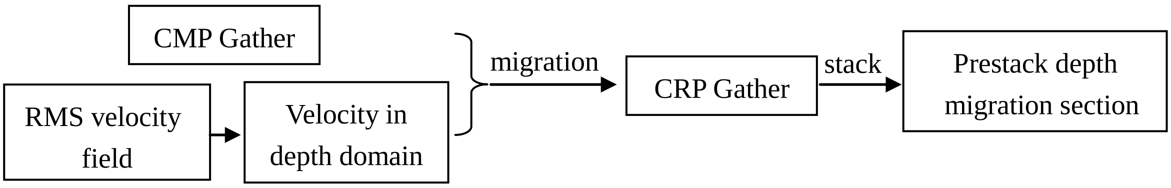

Supplement: Supplementary file 1 — Supplementary Information [file 41598_2017_15759_MOESM1_ESM.pdf]
